# The microbiome and mosquito vectorial capacity: rich potential for discovery and translation

**DOI:** 10.1186/s40168-021-01073-2

**Published:** 2021-05-18

**Authors:** Cintia Cansado-Utrilla, Serena Y. Zhao, Philip J. McCall, Kerri L. Coon, Grant L. Hughes

**Affiliations:** 1 Departments of Vector Biology and Tropical Disease Biology, Centre for Neglected Tropical Disease, Liverpool School of Tropical Medicine, Liverpool, UK; 2 Department of Bacteriology, University of Wisconsin-Madison, Madison, WI USA; 3 Department of Vector Biology, Liverpool School of Tropical Medicine, Liverpool, UK

**Keywords:** Microbiome, Vectorial capacity, Density, Competence, Biting, Extrinsic incubation period, Longevity, Mosquito, Symbiosis, Pathogen transmission

## Abstract

Microbiome research has gained considerable interest due to the emerging evidence of its impact on human and animal health. As in other animals, the gut-associated microbiota of mosquitoes affect host fitness and other phenotypes. It is now well established that microbes can alter pathogen transmission in mosquitoes, either positively or negatively, and avenues are being explored to exploit microbes for vector control. However, less attention has been paid to how microbiota affect phenotypes that impact vectorial capacity. Several mosquito and pathogen components, such as vector density, biting rate, survival, vector competence, and the pathogen extrinsic incubation period all influence pathogen transmission. Recent studies also indicate that mosquito gut-associated microbes can impact each of these components, and therefore ultimately modulate vectorial capacity. Promisingly, this expands the options available to exploit microbes for vector control by also targeting parameters that affect vectorial capacity. However, there are still many knowledge gaps regarding  mosquito–microbe interactions  that need to be addressed in order to exploit them efficiently. Here, we review current evidence of impacts of the microbiome on aspects of vectorial capacity, and we highlight likely opportunities for novel vector control strategies and areas where further studies are required.

Video abstract

**Supplementary Information:**

The online version contains supplementary material available at 10.1186/s40168-021-01073-2.

## Background

The 'microbiome' is a collection of microorganisms within or on an organism. In mosquitoes, the microbiome, which consists of bacteria, viruses, protozoans and fungi, profoundly alters host phenotypes. Acquisition and the composition of the microbiome are influenced by several abiotic and biotic factors, including host and microbial genetics [1–4] and the environment [5–7]. Therefore, microbiomes of mosquitoes can vary substantially between individuals, life stages, species and over geographical space [8, 9], and this variation likely contributes to differences in host phenotypes [10]. Similarly, the horizontal and vertical transmission routes that microbes exploit to colonise their host mean that mosquitoes reared in a laboratory setting have a vastly different microbiome compared to their field counterparts [11–13]. As such, undertaking studies with a field relevant microbiome has been challenging. Within the mosquito, microbes can invade and colonise different tissues, perhaps by intracellular routes [14], and the reproductive organs [15, 16] and salivary glands [17] appear to have the greatest diversity of microbes. Microbiota in the midgut or salivary glands have the potential to interact directly with pathogens whereas microbes residing in other tissues may indirectly affect vector competence. Microbes that reside in the gut or other tissues [18, 19] may also have relevance for other life history traits which influence vectorial capacity.

**Fig. 1:**
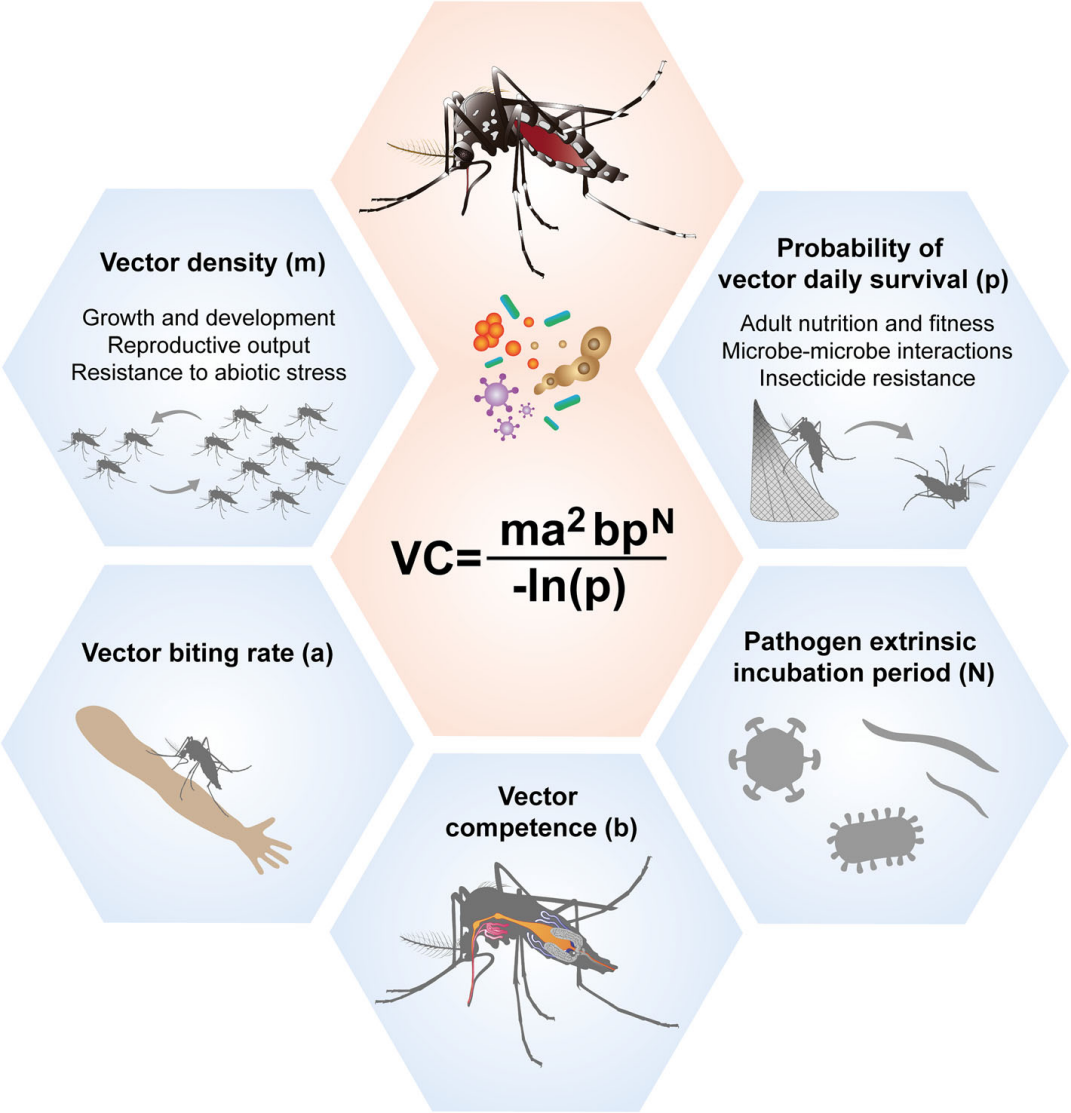
Vectorial capacity (VC) equation and the effects of the microbiome on mosquito vectorial capacity. The mosquito microbiome can modulate the five components of vectorial capacity. These components are vector density (m), vector biting rate (a), vector competence (b), pathogen extrinsic incubation period (N) and probability of vector daily survival (p). The microbiome can impact the probability of vector daily survival by modulating mosquito fitness, interacting with other microbes and affecting insecticide resistance. It can also affect vector density through effects on host growth, development and reproductive output and by modulating their resistance to abiotic stress

Vectorial capacity describes the ability of a population of vectors to transmit pathogens to a host and is represented by the vectorial capacity equation (Fig. 1). This was created by Garret-Jones in 1964 and represents the number of secondary cases of vector infection per unit of time given the introduction of an infectious individual into a naïve population [20, 21]. Pathogen transmission is modelled by the vectorial capacity equation, which is a vector-centric adaptation of the basic reproductive number (R_0_) equation [22]. The components of the vectorial capacity equation are the following: vector biting rate (a), vector density (m), probability of vector daily survival (p), vector competence (b) and pathogen extrinsic incubation period (N). An infected person gets bitten by *ma* vectors each day. Of these *ma* bites, only a proportion *b* is infectious to the vector, giving a total of *mab* vectors infected by the primary case. The proportion of vectors surviving the extrinsic incubation period is *p*^*N*^, so *mabp*^*N*^ vectors become infectious. Each of these infectious vectors then survives for an average time of 1*/−ln(p)*, and during this time, it bites people at the rate of *a* bites per day, making a total of *a/−ln(p)* bites. Thus, there are *mabp*^*N*^ infectious vectors arising from the primary case making *a/−ln(p)* infectious bites on susceptible hosts, resulting in the following vectorial capacity: *ma*^*2*^*bp*^*N*^*/−ln(p)*. Therefore, each component of the equation will have a certain impact on the ability of mosquitoes to transmit pathogens. As such, targeting any of these components could result in a reduction of pathogen transmission.

Some components of the vectorial capacity equation have traditionally received more attention than others by mosquito control efforts. Probability of daily survival and density have been targeted by adulticides and larvicides respectively, achieving significant reduction of vector-borne diseases, but the emergence of insecticide resistance and diverse non-target effects are compromising these strategies [23]. Vector competence has been the main focus of the design of novel vector control methods, such as release of *Wolbachia*-infected mosquitoes for population replacement, which has showed unprecedented success in dengue control [24]. However, little attention has been paid to other aspects of mosquito biology which can have equal or potentially greater effect on pathogen transmission [25–28]. In this sense, the great diversity of mosquito gut-associated microbes could offer new tools to target different components of vectorial capacity [29, 30]. However, in  order to leverage the microbiome for vector control, it is imperative to understand how such microbes  modulate vector biology. In this review, we compile and consider the evidence of the impact that the mosquito gut-associated microbiome has on particular components of the vectorial capacity equation. We also discuss other vector systems and are guided by what we can infer from other insect models. Finally, we draw from the substantial *Wolbachia* knowledge base when there is a lack of evidence on how gut-associated microbes influence traits relevant for vectorial capacity in mosquitoes.

## Influence of microbiota on vector competence (b)

All microbes that associate with vectors, including bacteria [31], viruses [32], fungi [33] or microsporidia [34] can modulate vector competence. Vector competence is fundamental to vectorial capacity since it determines the susceptibility of the mosquito to become infected by a pathogen, and the higher the vector competence, the higher the vectorial capacity. Gut-associated microbiota can interfere directly with pathogens through mechanisms such as lysis and biofilm formation [31] or indirectly by affecting intrinsic aspects of the vector that determine its vector competence, like midgut and salivary gland barriers [35–37] and the immune system [1, 38]. In addition, microbiota can potentially have other functions in pathogen transmission, since it may be transmitted to the mammalian host during feeding on the host [39]. The role of the gut microbiome in modulating vector competence for several pathogens has been well studied and reviewed extensively in mosquitoes [40–47] and other arthropod vectors [48–54], so we have focused our attention on the other components of the vectorial capacity equation.

## Influence of microbiota on pathogen extrinsic incubation period (N)

Little is known regarding how microbes influence the extrinsic incubation period (EIP), the time that it takes for pathogens to develop in the vector. This is distinct from vector competence, which concerns the ability of a vector to transmit a pathogen. The EIP affects vectorial capacity since it influences the number of infected mosquitoes that live long enough to become infectious and can vary depending on host and pathogen genetic factors and environmental conditions. There is evidence that *Wolbachia* infection can extend the EIP for DENV in *Aedes aegypti* [55, 56] and the authors argue that this may be due to the antiviral properties of *Wolbachia*, which delay the time that the virus titres reach an infectious threshold. Given that gut-associated microbiota modulate pathogens, it would be interesting to explore how the microbiota could be exploited to delay the EIP. Alternatively, microbiota that enhance the EIP could be potentially targeted to prevent a positive effect on pathogen transmission.

## Influence of microbiota on vector density (m)

Vector density is the number of vectors per host, and there is increasing evidence suggesting that the mosquito gut microbiota can modulate this facet of vectorial capacity. A sustained reduction in vector density leads to progressive population reduction in successive generations, resulting in reduced vectorial capacity. This principle was the cornerstone of many of the earliest vector control strategies, where breeding sites were eliminated or diminished, or treated with larvicides to reduce the number of vectors in a population. It is also the rationale behind more contemporary strategies such as the dissemination of insect growth regulators like pyriproxyfen [57] or the release of *Wolbachia*-infected male mosquitoes [58, 59]. Gut-associated microbes can influence vector density through the modulation of development, reproductive outputs, and resistance to abiotic stress.

### Growth and development

Recent work has elucidated the importance of microbes as a factor influencing growth and moulting of mosquito larvae into adults by regulating growth signalling and serving as a food source. Axenic (microbe-free) larvae fail to moult under normal environmental  conditions [60], and exhibit differential expression of genes relating to amino acid transport, hormone signalling, and metabolism compared to normal larvae [61]. Although some studies have produced larvae that developed without bacteria [62–64], the addition of living microbes appear to induce gut hypoxia and activation of growth-related signalling pathways that larvae require to achieve the critical size necessary for moulting [65–67]. In addition, gut hypoxia depends on bacterial density, as shown by *Ae. aegypti* larvae showing higher growth rates [67] and *Aedes albopictus* exhibiting enhanced adult emergence [68]. This indicates that the mechanisms responsible for regulation of host development under most conditions occur via microbial metabolism. In the absence of gut hypoxia [66], the larva fails to make adequate nutrient stores, so the mosquito is under microbial influence for accumulation of nutrient reserves that will take it into adulthood. Most mosquito species are detritivorous as larvae, using bacteria and other microorganisms as a food source [69], but predaceous species also consume microorganisms as food when prey are not available, so microbes can contribute to nutritional supply when food availability is a limiting factor [70, 71]. Reliance on gut hypoxia for signalling appears to be conserved across mosquito lineages, including detritivorous larvae from the Culicinae and Anophelinae subfamilies, and predaceous larvae of *Toxorhynchites amboinensis* [72], indicating that the role of larval gut microbiota in mosquito development is not limited to detritivory. Another condition that relies on the nutrients acquired during larval development is autogeny, which is the ability of some mosquito species to produce eggs without blood . Although both anautogenous and autogenous mosquito species rely on the larval microbiota for development, the autogenous *Aedes atropalpus* display limited rescue of development by some bacterial taxa when reared in monoculture, in contrast to its anautogenous relative *Ae. aegypti* [73]. This suggests that autogenous species may have more specific requirements for microbiota composition due to their reliance on larval nutrition and the absence of additional nutrient input from a blood meal. Gut microbes simultaneously regulate signalling and serve as a food source, and further study is required to identify any potential interactions of these dual functions and their impacts on vector life history.

Characterisation of microbiota effects on vector development begins with tracing impacts of individual microbial taxa and continues with the study of bacterial communities and their diversity. Although multiple microbial taxa individually support mosquito development [60], outcomes may differ according to nutrient conditions: *Ae. aegypti* larvae reared on *E. coli*, *Saccharomyces cerevisiae,* or *Chlamydomonas reinhardtii*, vary in their survival depending on their diet during rearing [67], while *Culex pipiens* reared on the human pathogen *Cryptococcus gattii* exhibit reduced larval survival and pupation compared to individuals reared on *S. cerevisiae* or yeasts isolated from wild *Cx. pipiens* and *Cx. theileri* [74]. Naturally occurring bacterial strains in the genera *Klebsiella* and *Aeromonas* are further able to support *Cx. pipiens* larval development from the first to second instar and are the most attractive to ovipositing females, but fail to produce surviving adults [75]. Particularly impactful microbes may also alter development even when they are not the sole occupant of the larval gut. For example, supplementation of conventionally reared larvae with a culture of *Asaia* accelerates *Anopheles gambiae* development; however, it is unknown whether this effect results from *Asaia* metabolism specifically, or merely from the increased bacterial density [76]. Diversity and community composition of the microbiota also impact development. Larvae reared in the presence of a combination of microbial isolates have higher pupation and survival rates than those reared in monoculture, indicating that a combination of cells of differing nutrient compositions and/or metabolic processes may have additive effects for larval nutrition [77]. In addition, antibiotic treatment, which decreases diversity and abundance of the gut microbiota, delays larval development by 2-4 days in *An. stephensi* [78]. However, supplementation of the disturbed microbiota with antibiotic-resistant *Asaia* restores development, suggesting that the roles of density and diversity in the gut microbiota’s modulation of host phenotype is complex and requires further testing.

### Reproductive output

The microbiome can also impact  mosquito reproductive output, which is the culmination of several physiological processes and population dynamics. It is influenced by sex ratio and mating behaviour, and results in egg production and hatching. Sex ratio is the number of males or females relative to the total number of emerged adults. The sex ratio of *Ae. aegypti* was shifted towards a male-biassed sex ratio when larvae were fed with bacteria or yeast [79], although the authors recognised that this could have been due to underfeeding. This may be the result of differences in larval metabolism and development between males and females, so further investigation is needed to understand the mechanisms underpinning this phenotype.

Mating starts with an encounter between individuals, the likelihood of which requires a certain threshold density of a population whose males and females can complete a full coordinated mating behaviour sequence successfully. There is evidence that these traits can be influenced by the gut microbiome. For example, studies in *Drosophila* indicate that larvae congregate in response to acetoin produced by the gut microbiome [80], leading to an increase in adult density over time. The absence of a gut microbiota in contrast leads to hyperactive adult behaviour [81] that is normalized by  the addition of *Lactobacillus*, which produces enzymes that influence neuronal pathways involved in locomotion [81]. Some mosquitoes mate in swarms, and variation in microbiota between swarms has also been observed [82], although further work is required to determine the cause and functional implications of these differences. After making an initial encounter a potential mate must have its identity and suitability as a fit mate confirmed before mating begins. In *Drosophila*, a greater number of matings were observed when males and females were reared on diets containing the same microbial consortia as opposed to diets with different microbial communities [83, 84]. Microbe-mediated changes in the levels or composition of sex pheromones and other mating cues could be responsible for this phenotype [85]. First, the production of hydrocarbons is regulated by the insulin signalling pathway, which is enhanced by *Wolbachia* in *Drosophila* [86]. Second, changes in the ratios of cuticular hydrocarbons affect mating recognition and sexual attractiveness of these and other flies [87–89]. Further investigation is required in order to disentangle the effects of the microbiota on host mating behaviour since this could affect genetic control strategies in vectors. For example, transgenic mosquitoes with enhanced immunity also have a modified microbiome and a mating fitness advantage compared to their wild type counterparts [90], potentially by microbiome-induced alterations of cuticular hydrocarbons. This resulted in wild-type male mosquitoes preferentially mating with genetically modified females and genetically modified males having a preference for wild-type females, thereby spreading the genetic modification into the population [90].

In addition to effects on sex ratio and mating behavior, egg production, oviposition, and hatching in insects are all affected by microbiota, and this impact on fecundity translates to changes in vector density. In general, fecundity in mosquitoes is governed by nutrients acquired during blood feeding, so blood digestion by adult females is necessary for egg production. A significant increase of microbe levels occurs after mosquitoes take a bloodmeal [91–93], and treatment of *Ae. aegypti* with antibiotics impedes digestion of blood proteins and consequently reduces egg production [94], suggesting that the microbiome contributes to blood digestion. Recently, it has been shown that sequential bloodmeals promote pathogen infection [95, 96], and it would be intriguing to determine the role of the microbiome in this phenotype. Recent studies also indicate that *Ae. aegypti* eggs laid in water containing bacteria hatch at a higher rate than those laid in sterile water [97] and female mosquitoes from many species preferentially oviposit in microbe-rich water [98]. Allelochemicals associated with bacteria have been identified [99], but the mosquito response can vary dramatically depending on its previous exposure to a particular chemical [100, 101]. Taking together, it is evident that gut microbes enhance mosquito fecundity and therefore the mechanisms that facilitate these phenotypes could be targeted to reduce vector density. As opposed to gut microbes, some *Wolbachia* strains seem to reduce female fecundity, egg hatch, and quiescent egg viability [102, 103], which results in a reduction of vector density and therefore vectorial capacity.

### Resistance to abiotic stress

Some vector species can survive (or are adapted to live) under adverse conditions, such as low humidity, brackish water or competitive environments, which permits colonisation of a broader range of environments. Resistance of mosquito eggs to desiccation is variable among species, and three main factors drive this variability: chitin content, egg volume and shell density [104]. Evidence that gut-associated microbiota regulate two enzymes involved in chitin synthesis (GFAT and CHS2) in *An. gambiae* [37] suggests the potential for the microbiome to influence resistance to desiccation. Once eggs have hatched, larvae have to persist in their aquatic environment. Whilst most mosquito species breed in fresh water, *Culex sitiens* and *An. sundaicus* survive in brackish water [105]. In general, the microbiome can confer resistance to salinity in plants and animals [106, 107], suggesting similar advantages could be conferred by gut-associated microbes to their mosquito hosts. Mosquito larvae in natural environments also occur within food webs that include both interspecific and intraspecific competitors and predators. The influence of the microbiome on larval competition is still to be determined, but *Wolbachia* infection has been shown to cause a density-dependent effect on larval survival [108]. Microbes that protect mosquitoes against abiotic stresses would be good candidates for paratransgenesis as this trait would likely facilitate their spread and persistence in the mosquito population.

## Influence of microbiota on probability of vector daily survival (p)

The probability of daily survival is the chance that a vector survives each day, and pathogens with longer EIPs may be particularly sensitive to this parameter. The microbiome has the potential to affect survival by altering adult nutrition and fitness, interacting with other microbes, and modulating insecticide resistance.

### Adult nutrition and fitness

The microbiome can impact insect survival by affecting host fitness, nutrition, homeostasis, and metabolism of their host. One indicator of mosquito fitness (among many others) is body size, and in general microbiota enhance development and size of mosquitoes. For example, *An. gambiae* and *An. stephensi* supplemented with *Asaia* have shown increased growth rates [76]. Similarly, when *An. coluzzi* mosquitoes were reared on three distinct diets, larger mosquitoes where seen to harbour a greater bacterial load [109]. Mosquito larvae fed solely with either bacteria or yeast still developed, although were smaller than their counterparts fed on food sources [79], suggesting that microbes alone provide some sustenance for the insect. Smaller mosquitoes are more susceptible to environmental stressors and thus have a reduced chance of survival [110]; therefore, microbe stimulation of nutrition can influence vector population dynamics.

Adult mosquitoes obtain their nutrients from two food sources, sugar and blood, and the microbiome plays an important role in food digestion and nutrient provision. *Enterobacteriaceae* is the most active family of the gut microbiota of *Ae. albopictus* at assimilating fructose, a major sugar component of nectar [111] and this sugar is used by bacteria as an energy source to produce other nutrients for the mosquito host. The impact of gut-associated microbiota on nutrition has also been studied in model insects, and results in these systems could shed insights into mechanisms occurring in mosquitoes. Examples include complementation of vitamins missing from the diet in other hematophagous insects [112] and *Drosophila* [113], and alteration of expression of genes involved in energy storage in *Riptortus pedestris* [114].

In *Ae. aegypti* [115] and *An. arabiensis* [116], disturbance of gut homeostasis resulted in a shortened lifespan, so inducing microbiome dysbiosis in vectors may be explored as a novel control strategy. There is precedent for microbial-based life-shortening approaches, with modelling and empirical evidence suggesting some strains of *Wolbachia* can reduce pathogen transmission due to their effects on longevity [117–119] and density [120]. However, this strategy was not pursued after it became apparent that *Wolbachia* interfered with pathogen development in the vector, and hence, population replacement could be undertaken by that route instead. Microbiome-mediated alterations in metabolites in the host can also lead to different survival outcomes. A recent study demonstrated that bacteria which lowered methionine content of food extended *Drosophila* host lifespan [121]. Although this was tested in flies, methionine has been shown to act as a larvicide against several mosquito species such as *An. quadrimaculatus*, *Ae. albopictus* and *Cx. tarsalis* [122], suggesting that similar processes could occur in mosquitoes. Another study in *Drosophila* showed that the production of lithocholic acid by the adult gut microbiota elongated host survival through upregulation of genes involved in glucose homeostasis [123], offering a potential target in the host to shorten lifespan. The insulin growth factor signalling pathway is central to regulation of lifespan [124–126], and can be impacted by bacterial metabolism in mosquitoes [66], although the mechanisms are unknown.

Host-microbe symbioses are complex and are influenced by host physiology, microbial composition and the timing of infection. The lifespan of *An. coluzzii* is extended with exposure to doxycycline but shortened with azithromycin [127], suggesting that changes in microbiome composition are driving this phenotype, although direct effects from the antibiotic need to be considered. Similarly, axenically reared or antibiotic-treated *Drosophila* had reduced lifespans, but if flies were exposed to bacteria in their first week as adults, their lifespan was similar to their conventionally reared counterparts [128]. In contrast, a study that compared axenic *D. melanogaster* with gnotobiotic flies infected with *Acetobacter pomorum* found no differences in survival. However, axenic flies had greater glucose levels and lower oxygen consumption, suggesting a potential overall slowing of respiration [129]. These findings indicate that host changes associated with microbiota may manifest as intermediate phenotypes rather than detectable changes in lifespan and thus studies that measure overall fitness outcomes may miss subtle effects of the microbiota. Further work is needed to identify which affected host functions impact longevity, and whether similar longevity phenotypes may obscure other trait differences. Host-microbe interactions become even more complex when some members of the microbiome shift from a commensal to a pathogen status and vice versa [130]. This can happen due to temperature, presence of pathogens, and other unknown factors [131, 132]. Such transitions of status and the broad range of possible complexities of host-microbe interactions should not be ignored when considering basic research questions and ultimately when considering microbiome control strategies.

### Microbe–microbe interactions

The diverse microbes that reside within insects may interact with pathogens that are detrimental to the host, making the vector either more resistant, tolerant, or susceptible to infection and thus impacting lifespan. For example, *Rickettsia*, an endosymbiont of whiteflies, reduces the density of pathogenic *Pseudomonas* resulting in an extended lifespan for its host [133]. In contrast, the infection of mosquitoes with the pathogenic fungus *Beauveria bassiana* causes microbiome dysbiosis and over-proliferation and translocation of *Serratia marcescens* from the gut to the hemocoel, eventually killing the insect [134]. Microbes can benefit from each other, like symbiotic bacteria and yeast in *Drosophila* [135], but they can also exclude one another, like *Enterobacteriaceae* and *Serratia* [136] or *Asaia* and *Wolbachia* [137] in mosquitoes. In flies and mosquitoes, microbiota interactions with *Wolbachia* occur but these do not influence the ability of *Wolbachia* to block pathogens [138, 139]. However in general, these complex microbial interactions determine microbiome composition and colonisation of the host [11, 140], influencing host physiology and lifespan [141] and therefore the effectiveness of microbial control of mosquitoes [142].

### Insecticide resistance

Gut-associated microbiota may also indirectly affect mosquito lifespan by mediating resistance to insecticides. Evidence is emerging that mosquitoes with differing resistance status have distinct microbiomes [143, 144], but further work is required to investigate the causality and the mechanisms underpinning these associations. *Streptococcus*, *Pseudomonas*, *Klebsiella,* and *Pantoea* correlated with insecticide resistance in *An. arabiensis* [145], *An. albimanus* [146, 147] and *An. stephensi* [148]. *Wolbachia* has also been associated with insecticide resistance in *Culex pipiens* [149]. Detoxifying symbionts in the gut microbiome have been shown to confer insecticide resistance in other insects like wasps [150], honeybees [151] and insect pests [152]. Although the mechanisms have not been described in mosquitoes, the ability of some of these bacteria to degrade insecticides [146] provides a possible explanation. Additionally, bacteria present in the soil may become resistant to insecticides due to chronic exposure [153] and these bacteria may colonise insects, either transiently or stably. A more complete understanding of the role of the microbiome on insecticide resistance will enable the development of strategies to mitigate the emergence of resistance and extend the longevity of currently used formulations.

## Influence of microbiota on vector biting rate (a)

Vector biting rate is the average number of times that a vector bites per unit of time and can be modulated by the microbiome by impacting feeding behaviour and host preference. An increased biting rate leads to a higher vectorial capacity, since the vector has more opportunities to acquire and transmit pathogens. Feeding behaviour is disrupted in *Ae. aegypti* by *Serratia* [136] and in *Anopheles* mosquitoes when exposed to heat-killed *E. coli* [154] or *Chromobacterium* [155]. Microbiota also have the potential to affect host-seeking behaviour through modulation of their chemosensory system. In *D. melanogaster*, symbionts determine larval pheromone preference [80] and affect the adult olfactory system, influencing food choice [156–158]. Additionally, gut bacteria are known to modulate expression levels of vitellogenin genes in the true bug, *Riptortus pedestris* [114], and in *Ae. albopictus*, vitellogenin expression regulates host-seeking behaviour [159]. Therefore, the ability of the microbiome to impact host seeking behaviour, possibly through modulation of vitellogenesis, should be further investigated.

## Conclusions

There is emerging evidence that the microbiome of vectors can influence many traits important for vectorial capacity. At the same time, many studies highlight the complexities of microbial communities and variability of the microbiome in mosquitoes. Attempts to disentangle this complexity often examine the effect of a specific microbe on the host, such as those that exploit mono-axenic gnotobiotic infections; however, it is unclear if these findings translate to mosquitoes with a complete microbiome consisting of many microbes. Additionally, applied strategies need to be effective in hosts with divergent microbiomes which mosquitoes possess in the field so understanding microbial interactions is integral. Other challenges for the scientific community to solve include moving beyond simple descriptions of the microbiome of distinct mosquito cohorts or mosquitoes with differing treatments to validating the microbes or microbial consortia that are the causal agents of host phenotypes, and the eventual elucidation of the mechanisms responsible for those interactions. Much can be learned from other research areas where the complexity of microbial community composition is also a challenge [160–164]. Advances in omics technologies can be used to disentangle this complexity, but this can also be addressed by grouping microorganisms with common life history and interspecific relationships [165], which can then be linked to effects on the host and then vectorial capacity. Ultimately, the development of sustainable strategies to modulate vectorial capacity by introducing microbes into wild mosquito populations will require a thorough understanding of microbiome acquisition and the factors controlling its composition and abundance. Only then can the full potential of the microbiome for vector control be realised.

## Data Availability

Not applicable
